# In the Aftermath of School Victimization: Links Between Authoritative School Climate and Adolescents’ Perceptions of the Negative Effects of Bullying Victimization

**DOI:** 10.1007/s10964-021-01516-x

**Published:** 2021-10-19

**Authors:** Kevin A. Gee, Misha D. Haghighat, Tseng M. Vang, North Cooc

**Affiliations:** 1 School of Education, University of California, Davis, CA USA; 2 Human Development Graduate Group, University of California, Davis, CA USA; 3 Department of Human Ecology, University of California, Davis, CA USA; 4 Department of Special Education, The University of Texas at Austin, Austin, TX USA

**Keywords:** Authoritative school climate, Bullying victimization, Negative feelings; Ordinal logistic regression, National Crime Victimization Survey

## Abstract

Although authoritative school climate—strict, yet fair enforcement of rules alongside strong adult support—is associated with lower rates of bullying victimization, less is known about whether it influences how negatively adolescents feel after being victimized at school. Further, it is unclear whether boys and girls respond differently to an authoritative climate. Identifying ways that schools can reduce negative feelings after being bullied is important given the long term psychological ramifications of bullying that, if left unaddressed, can extend into adulthood. To address these gaps, this study examined whether authoritative school climate related to how negatively adolescents felt about their schoolwork, relationships, physical health and self-perception after being bullied. Differences between boys and girls were also investigated. Analyses were conducting using national data from the 2017 School Crime Supplement on a sample of 1,331 adolescents aged 12 to 18 years (*M*_*age*_ = 14.3 years; 59% girls). Findings from a set of ordinal regression models with a robust set of student, parent and school controls demonstrated that adolescents in more supportive schools were less likely to report that bullying victimization negatively impacted their schoolwork and feelings about themselves. Similar results were found for girls but not boys. By investing in supportive school climates, schools can be potentially transformative places where adolescents, especially girls, can feel more positively about themselves despite being bullied.

## Introduction

Previous research has established that schools with stronger authoritative climates, those based on strict yet consistent rules coupled with support from caring adults, can reduce overall rates of bullying victimization during adolescence (Gregory et al., 2010). Yet, less is known about whether an authoritative climate can also reduce negative feelings adolescents can have about their daily social lives in multiple domains such as schoolwork, relationships, physical health and feelings about one’s self after being bullied. Understanding whether authoritative school climates can effectively counteract these negative feelings may alleviate the longer-term consequences of bullying victimization that can extend into adulthood (Wolke & Lereya, 2015). Further, given that girls disproportionately experience negative psychological effects of bullying victimization (Ledwell & King, 2013), it is important to determine whether authoritative climates can promote girls’ wellbeing after being victimized. To address these gaps, this study analyzes data from the 2017 School Crime Supplement (SCS) of the National Crime Victimization Survey (NCVS) to investigate the link between authoritative school climate and how bullying victimization affected adolescents’ (a) schoolwork, (b) relationships with friends and family, (c) feelings of self, and (d) physical health. Gender differences are also investigated.

### Bullying in Adolescence

Bullying victimization refers to ongoing and repeated aggressive acts against others that occur within an asymmetric power relationship (Olweus, 2013). Bullying is typically classified into key subtypes including physical (e.g., inflicting physical harm), verbal (e.g., using derogatory words or insults) and relational (e.g., spreading rumors) (Kennedy, 2020). Among adolescents, bullying is commonplace, with nearly 1 in 5 students aged 12 to 18 reporting some form of bullying victimization. Moreover, girls report higher rates of bullying victimization versus males (26% versus 19% in 2019; National Center for Education Statistics, 2020). Bullying can negatively impact children’s and adolescents’ physical health (e.g., self-harm), mental health (e.g., internalizing problem behaviors), social relationships, and academic achievement (Wolke & Lereya, 2015). Given these negative consequences, identifying strategies to address and prevent bullying in childhood and adolescence has emerged as a central concern especially for schools. Schools themselves, particularly their overarching climates consisting of norms, values, and social relationships (Thapa et al., 2013), can be highly influential in mitigating bullying and its consequences.

### Authoritative School Climate Theory: School Disciplinary Structure and Student Support

Authoritative school climate theory, also referred to as authoritative discipline theory (Gregory et al., 2010), is grounded in the concept of *authoritative parenting* which posits that clear boundaries alongside support can positively influence children’s outcomes (Baumrind, 1968). Two core concepts underlie authoritative climate theory: school disciplinary structure and student support. Each has links to bullying and bullying-related outcomes, including adolescents’ socioemotional wellbeing.

#### School disciplinary structure

School disciplinary structure refers to the degree that schools consistently and fairly enforce school rules (Gregory et al., 2010). It has been linked with reductions in depressive symptoms, anxiety, and conduct problems (Hendron & Kearney, 2016). Previous research indicates that in schools where youth are aware of school rules and teachers actively intervene on behalf of bullying victims, youth report a lower incidence of bullying (Låftman et al., 2017). However, youth who believe that school rules are inequitable face a heightened risk of bullying victimization (Kupchik & Farina, 2016). Beyond the incidence of bullying, research also shows that adolescents who believe that school rules are fair are less likely to fear being harmed at school or to avoid school for fear of being attacked (Mulvey et al., 2018). As such, victimized youth may feel emotionally and physically safer in schools that enforce consistent and fair rules, especially since they know that schools will hold bullies accountable for engaging in harmful acts. While disciplinary structure can potentially reduce negative feelings of fear and lead to enhanced safety, whether these effects also extend to the feelings that adolescents have after being victimized remains open for further empirical investigation.

#### Student Support

Student support refers to the presence of a caring adult, such as a teacher or school staff member who treats students with warmth and respect (Konold et al., 2014). Adult support has been positively associated with school outcomes such as motivation, academic achievement, and sense of belonging (Kiefer et al., 2015). Teacher support, in particular, is linked to reductions in a range of negative outcomes including drug abuse (Earnshaw et al., 2014) and suicidal ideation (Madjar et al., 2018). Youth who perceive adult support as more positive can feel more included and safer at school, which can be particularly important for youth who have experienced bullying victimization. Not only can teacher support reduce students’ fear of future bullying attempts (Baek et al., 2019), but when supportive teachers and school staff proactively prevent or reduce bullying, emotional/relational and physical bullying victimization declines and students’ likelihood of intervening in bullying incidents increases (Espelage et al., 2014). Relatedly, students who also trust their teachers have an increased sense of safety at school (Williams et al., 2018). Supportive teachers can also promote adolescents’ wellbeing by helping them alter how they appraise stressful events. After experiencing a stressful event such as victimization, students could appraise and re-appraise that stress less negatively if they know that adult support is available to help them cope with stress (Davidson & Demaray, 2007). Yet, further research is needed to pinpoint whether support can also help promote adolescents’ emotional and physical wellbeing after being victimized.

### Gender Differences in Response to Authoritative School Climate

Although the literature on school disciplinary structure and social-emotional adjustment by gender is limited, a handful of studies indicate that school disciplinary structure may lead to gender differences in wellbeing. For boys, authoritative school climate is negatively associated with internalizing and externalizing problem behaviors, while for girls, school climate is associated only with externalizing problems (Kuperminc et al., 1997). This pattern is also consistent with findings that school disciplinary structure, when considered separate from other aspects of school climate, is only significant in reducing boys’ internalizing problem behaviors (Suldo et al., 2012).

Previous research has indicated that girls may be especially susceptible to incidents of bullying victimization (Pontes et al., 2018) given greater propensity for identity-based victimization (i.e., victimization based on one’s gender identity; Price et al., 2019). Adult support may be particularly critical for girls who may be generally more comfortable asking for support from teachers than boys (Schenke et al., 2015). Thus, girls may also be more likely to report bullying to adults (i.e., parents and teachers; Blomqvist et al., 2020) and seek help to resolve bullying incidents. However, given that girls are more likely to seek support, they may experience negative impacts when supports are not provided. In fact, in one study, girls who did not receive teacher support were more likely to condone violent activities (Pérez-Martínez et al., 2021).

While teacher support may be critical for youth, there is mixed evidence about whether support benefits girls more than boys. Typically, girls are more likely to engage in help-seeking behaviors than boys. A study of help-seeking behaviors in youth (ages 9–14) from Scotland demonstrated that girls were more likely to report that social support was the most effective method for reducing bullying episodes and uplifting their mood (Hunter et al., 2004). Relatedly, a study of victimized adolescents in China who had faced verbal belittlement and/or physical attacks found that teachers and peers were more effective for buffering depression in girls (Guo et al., 2020). In contrast, other evidence shows that despite having stronger connections with teachers, girls still reported higher levels of depression than their male peers (Price et al., 2019). Moreover, even with stronger support, girls who reported the highest levels of bullying discrimination also reported high depression ratings (Price et al., 2019). This depression could counteract the increased levels of emotional regulation, coping and emotional stability that support can provide; as a result, females could experience more negative feelings about being victimized relative to boys even if they are in schools with similarly strong authoritative school climates. These divergent findings may be due to different cultural contexts of these studies. Youth in China may have a greater sense of respect for their teachers and as such report more favorable teacher and student relationships versus youth from the United States (Bear et al., 2014). As a result, teacher support may have had more of an impact in reducing the negative emotional effects of bullying victimization for girls from China. Finally, for both boys and girls, support from family and/or friends rather than teacher support alone was more effective in buffering youths’ negative emotions from bullying (Hunter et al., 2004). In fact, research has shown that support from professionals (e.g., a teacher or school counselor) was neither associated with boys’ nor girls’ mental health (Noret et al., 2020) and, thus, adult support at school could be unrelated to both girls’ and boys’ negative feelings after being bullied.

## Current Study

Researchers have seldom examined whether stronger authoritative school climates can influence students’ negative feelings after being bullied. A more robust understanding is needed of how the structural and malleable conditions of schools can promote adolescents’ emotional resiliency after being bullied. Further, examining gender differences helps draw clearer distinctions about for whom authoritative climate can be most influential and has implications for ways schools may need to support girls and boys differently. Accordingly, this current study is guided by two research questions: (1) How does authoritative school climate relate to how negatively adolescents feel about their schoolwork, relationships, physical health and self-perception after being bullied? (2) Does this relationship differ for boys and girls? Given the extant research base linking stronger authoritative climates to enhanced socioemotional wellbeing, this study hypothesizes that adolescents in schools with stronger structure and support will also report that they experienced fewer negative effects of bullying on their outcomes, especially on their feelings of self (Hypothesis 1). Effects on feelings of self relative to other outcomes (e.g., physical health) are expected to be the strongest given the prior evidence that both structure and support can make adolescents feel more emotionally secure. Given the mixed evidence on gender differences in response to authoritative climates, this study does not propose a hypothesis regarding how climate can differentially affect boys relative to girls.

## Methods

### Dataset and Sample

This study used data from the 2017 School Crime Supplement (SCS) of the National Crime Victimization Survey (NCVS; United States Bureau of Justice, 2020). The SCS is administered biannually to 12 to 18 year olds from households selected to complete the NCVS. Households are selected for the NCVS based on addresses from the most recent census. SCS respondents attend both public and private schools (6th to 12th grades) throughout the United States. The supplement is administered in-person using computer-assisted personal interviewing (CAPI). The SCS asks adolescents about their experiences with, and perceptions of crime and safety at school, such as hate-related incidents, fear of victimization at school, and the presence of drugs and weapons.

The dataset is well suited for this analysis because it includes measures used in prior research on authoritative climate theory (Gee & Cooc, 2019) and captures adolescents’ negative feelings after being victimized. Because the data are publicly available and de-identified, the research was determined not to involve human subjects and therefore, Institutional Review Board review was not required.

The 2017 SCS sample includes adolescent respondents (*N* = 6199) with valid survey interviews (students who did not attend school, were homeschooled or not in grades 6–12 were excluded). From the full sample, the study used an analytic subsample of adolescents (*n* = 1331) that reported being victimized during the past school year. Missing data (described in the appendix) was handled using multivariate imputation by chained equations (MICE; Royston, 2004).

### Measures

#### Negative impacts of bullying victimization

The study outcome captures how negatively (coded in 3-categories: not at all/not very much, somewhat, or a lot) bullying affected students’ (a) school work, (b) relationships with friends and family, (c) feelings about self, and (d) physical health (e.g., injuries, headaches, etc.). The outcome was treated as an ordered categorical variable given that students’ responses were on a Likert scale that is ordinal rather than interval (i.e., differences between adjacent response categories are not equal).

#### Disciplinary structure

Consistent with Fisher et al. (2018), factor analysis was used to generate a continuous factor score (*M* = 0; *SD* = 1) capturing a school’s disciplinary structure. Schools with higher disciplinary scores have higher factor scores. The scores were constructed using the full sample of both victimized and non-victimized adolescents and the original scores were retained when analyzing the subsample of victimized adolescents. The factor scores were based on four items capturing the extent to which adolescents agreed, on a 4-point scale from 1 (*strongly agree*) to 4 (*strongly disagree*), that (a) their school’s rules were fair, (b) the punishment for breaking rules was the same for everyone, (c) rules were strictly enforced, or (d) students knew the punishment if rules were broken. Ordinal reliability for the items was 0.87. Factor analysis based on a polychoric correlation matrix yielded one factor (eigenvalue = 2.75) with each item loading high on this one factor (loadings were > 0.78).

#### Student support

The measure of student support was based on a continuous factor score (*M* = 0; *SD* = 1) using factor analysis on four items available in the SCS that have been used in prior research to measure student support (Cornell et al., 2015). Three of the items captured the extent to which adolescents agreed, on a 4-point scale from 1 (*strongly agree*) to 4 (*strongly disagree*), that there was a teacher or adult at school who (a) really cared about them, (b) listened to them when they had something to say, or (c) told them when they did a good job. The other item asked adolescents to report on the same 4-point scale the extent to which teachers at their school treated students with respect. Ordinal reliability for the items was 0.91. Factor analysis based on a polychoric correlation matrix yielded one factor (eigenvalue = 2.80) with each item loading high on this one factor (loadings were > 0.94).

### Control Variables

This study controlled for a robust set of student, parent and school characteristics that could be confounded with both authoritative school climate and the outcome measures. These controls, based on student self-reports, have been included in prior studies that have used the SCS to study school bullying victimization (Gee & Cooc, 2019) and reflect socio-ecological factors underlying bullying victimization (Espelage, 2014). Further, these socio-ecological factors capture aspects of individuals, their parents and characteristics of their schools that exert influence on how adolescents develop (Governale & Garbarino, 2020).

#### Bullying victimization type

Victimization type consisted of six categories: (a) physical (they were pushed, shoved, tripped or spit on); (b) social (they had been made fun of, had rumors spread about them, or had been excluded); (c) threatened with harm; (d) coerced; (e) had property destroyed; or (f) called hate-words.

#### Bullying victimization frequency

How often victimization occurred in the past school year was reported in four categories: one day, two days, three to 10 days, or more than 10 days.

#### Fear and avoidance of school

Continuous factor scores (*M* = 0; *SD* = 1) were constructed capturing school fear and avoidance of school. Fear was based on two items asking students how often (from *never* to *most of the time*) that someone will attack or harm them in (a) school building or on school property; or (b) on a school bus or on the way to and from school. Reliability of the items was 0.94. Factor analysis yielded one factor (eigenvalue = 1.88). Avoidance was based on twelve items. Ten of the items asked students whether they stayed away from certain places at (e.g., cafeteria) or on their way to school (e.g., school bus) because they thought someone would attack or harm them. Three additional items asked students whether they (a) avoided activities, (b) avoided classes or (c) stayed home because they thought someone might harm or attack them. Ordinal reliability of the items was 0.98. Factor analysis yielded one factor (eigenvalue = 8.94).

#### School security features

The total number of school security features was included. Students reported whether their school had: (a) security guards or assigned police officers; (b) school staff or adults supervising the hallway; (c) metal detectors, including wands; (d) locked entrance or exit doors; (e) a requirement for visitors to sign in and wear badges; (f) locker checks; (g) a requirement for students to wear identification; (h) one or more security cameras; (i) a student code of conduct.

#### Caring peer index

A continuous caring peer factor score (*M* = 0; *SD* = 1) was based on three items capturing the extent to which adolescents agreed, on a 4-point scale from 1 (*strongly agree*) to 4 (*strongly disagree*), that a student at school (a) really cared about them, (b) listened to them when they had something to say, or (c) believed they would be a success. Ordinal reliability of the items was 0.99. Factor analysis yielded one factor (eigenvalue = 2.37) with the three underlying items loading high on this one factor (loadings were > 0.83).

#### Grades

Students’ self-reported grades across all subjects in the present school year were included in three categories: mostly A’s, mostly B’s, and mostly C’s or below.

#### Extracurricular activities

The number of school extracurricular activities students participated in were included. Students reported whether they participated in four extracurricular activities: academic clubs, athletics, performing arts or student government.

#### Public school and school level

Students reported whether they attended public or private school and their grade level (grades 6 through 8 were combined and coded as 1 = middle school, 0 = high school).

#### Guns, physical fights and gangs

To capture the schooling environment, this study included dichotomous indicators for whether the student knew if another student brought a gun to school, whether they engaged in one or more physical fights at school and whether gangs were at their school.

#### Demographic characteristics of students

The study controlled for a student’s age (in years), gender (coded as 0 = male, 1= female) and race and ethnicity (coded into 5-categories: White, Black, Hispanic, Asian or another race or ethnicity).

#### Parental education level

Parents’ highest grade level attained was coded in three categories: elementary, middle school, or high school or above.

### Data Analysis Plan

Ordinal logistic regression was used to estimate whether the authoritative disciplinary predictors were linked to a lower probability that adolescents reported negative effects of bullying victimization on their outcomes. The model fit to data for child *i* was as follows:

$$\begin{array}{l}{{{\mathrm{logit}}}}\left( {P(Y_i \le j)} \right) = \alpha_j - \beta _1(DisciplinaryStructure)_i - \beta _2(StudentSupport)_{i} - \mathop {\sum }\limits_{q = 0}^Q \gamma_{q} x_{i}\end{array}$$

where *j* is the ordered levels for each outcome (e.g., 3 levels of how negatively bullying victimization affected their outcomes: not at all/not very much; somewhat; a lot). The outcome, logit (*P*(*Y*_*i*_ ≤ *j*)), is the log odds of being in category *j* or below (e.g., a lot) versus in the rest of the categories above *j* (e.g., somewhat or not at all/not very much). *γ*_*q*_ captures the effects of the controls *x*_*i*_ such as student demographic characteristics. In this model, *β*_1_ and *β*_2_ represent the parameters of interest. This study adopted a conventional level of significance (α = 0.05) to test the null hypothesis that there was no relationship between either support or structure on the stated outcomes. Using these estimates, predicted probabilities were calculated of being in each level of *j* and then plotted to show how those probabilities changed based on the values of the disciplinary and student support indices. Models were fit for the sample overall and then separately for each gender subgroup.

Analyses were conducted using the survey commands in Stata 15.1 and incorporated appropriate weights to account for the complex sampling design of the *NCVS* and *SCS* survey data. Given that multiple imputation was used to handle missing data, models were fit across 25 imputed datasets and the results were pooled together. Standard errors were estimated using Taylor linearization. Finally, an important limitation of the data to note is that the *SCS* data did not have school-level identifiers, thus clustering at the school-level in the statistical models was not included.

Before fitting full models across each imputed datasets, the parallel lines (or proportional odds) assumption was tested to determine if the effects of the main authoritative climate predictors were equal across each of the outcome categories, overall and by each gender subgroup. A series of diagnostic models were fitted on non-imputed data in Stata 15.1 using the gologit2 command with the auto option (Williams, 2005) that uses a Wald test to determine whether the coefficient estimates were the same across the different categories of each outcome. All models yielded insignificant test statistics (*p* > 0.05) suggesting that the parallel lines assumption was met.

## Results

### Descriptive Statistics

Table 1 presents weighted means and standard deviations on the analytic sample overall and disaggregated by gender. In terms of adolescents’ demographic characteristics, the sample was predominately White (61%) followed by Hispanic (18%) and Black (16%), and a majority of adolescents (66%) had parents with some college education or above. The most frequent response for each outcome overall and in each gender subgroup was that bullying victimization did not negatively or have very much of a negative impact on adolescents’ school work, relationships, feelings about self or physical health. Bullying victimization impacted negative feelings about self somewhat or a lot for 26% of adolescents overall, 31% for girls and 19% for boys. Relative to girls, fewer boys responded that relationships with friends and family were negatively impacted somewhat or a lot by bullying victimization (9% of boys versus 17% of girls reported “somewhat” and 3% of boys versus 6% of girls reported “a lot”). Similarly, fewer boys tended to report that bullying victimization had negatively impacted their physical health either somewhat or a lot.

**Table 1:** Weighted descriptive statistics for a sample of adolescents who experienced bullying victimization in the 2016-7 school year, overall and by gender (2017 School Crime Supplement to the National Crime Victimization Survey; n (unweighted) = 1032)

	Overall		Girls		Boys	
	Mean or proportion	*SD*	Mean or proportion	*SD*	Mean or proportion	*SD*
Negative effects of bullying on:						
School work						
Not at all/Not very much	0.82	0.38	0.82	0.39	0.82	0.38
Somewhat	0.14	0.34	0.13	0.34	0.15	0.35
At lot	0.04	0.20	0.05	0.22	0.03	0.18
Relationships						
Not at all/Not very much	0.82	0.38	0.78	0.42	0.88	0.32
Somewhat	0.13	0.34	0.17	0.38	0.09	0.28
At lot	0.05	0.21	0.06	0.23	0.03	0.18
Feelings about self						
Not at all/Not very much	0.74	0.44	0.70	0.46	0.80	0.40
Somewhat	0.16	0.37	0.18	0.38	0.13	0.34
At lot	0.10	0.30	0.13	0.33	0.06	0.24
Physical health						
Not at all/Not very much	0.87	0.34	0.84	0.37	0.90	0.30
Somewhat	0.10	0.30	0.11	0.32	0.08	0.28
At lot	0.03	0.18	0.04	0.20	0.02	0.14
Authoritative school climate						
Disciplinary structure index	−0.28	1.02	−0.33	1.09	−0.19	0.93
Student support index	−0.25	1.11	−0.22	1.04	−0.29	1.21
Bullying victimization type						
Physical victimization	0.26	0.44	0.18	0.39	0.37	0.48
Social victimization	0.94	0.24	0.97	0.17	0.89	0.31
Threatened with harm	0.19	0.39	0.14	0.35	0.25	0.43
Coercion	0.09	0.29	0.07	0.25	0.12	0.32
Destroy property	0.07	0.25	0.06	0.24	0.07	0.26
Hate-related words	0.24	0.43	0.23	0.43	0.25	0.43
Bullying victimization frequency						
One day	0.31	0.46	0.28	0.46	0.36	0.48
Two days	0.18	0.39	0.19	0.40	0.18	0.38
Three to 10 days	0.29	0.45	0.29	0.46	0.29	0.45
More than 10 days	0.21	0.41	0.23	0.43	0.18	0.38
Fear of school index	−0.28	1.39	−0.35	1.53	−0.18	1.18
School avoidance index	0.27	1.15	0.32	1.17	0.20	1.11
Number of school security features	5.99	1.24	5.97	1.30	6.02	1.17
Caring peer index	−0.13	1.08	−0.10	1.08	−0.16	1.09
Self-reported grades						
Mostly A’s	0.40	0.49	0.46	0.50	0.31	0.46
Mostly B’s	0.40	0.49	0.38	0.49	0.44	0.49
Mostly C’s or below	0.20	0.40	0.16	0.37	0.26	0.44
Number of extracurricular activities	1.12	0.94	1.17	0.97	1.05	0.91
Attends a public school	0.93	0.26	0.93	0.26	0.93	0.26
In middle school (grades 6–8)	0.52	0.50	0.50	0.50	0.55	0.50
Students brought guns to school	0.08	0.27	0.09	0.29	0.07	0.25
Involved in physical fights at school	0.13	0.34	0.06	0.24	0.24	0.42
Gangs at school	0.19	0.39	0.18	0.39	0.20	0.40
Age (in years)	14.30	1.80	14.34	1.84	14.25	1.75
Male	0.41	0.49	–	–	–	–
Race and ethnicity						
White, non-Hispanic	0.61	0.49	0.63	0.49	0.59	0.49
Black, non-Hispanic	0.16	0.36	0.16	0.37	0.15	0.36
Hispanic	0.18	0.38	0.17	0.38	0.17	0.38
Asian	0.02	0.13	0.01	0.11	0.02	0.15
Another race or ethnicity	0.04	0.20	0.03	0.17	0.06	0.23
Parental education level						
Elementary (5th grade or below)	0.01	0.08	0.01	0.08	0.01	0.08
Middle (6th through 8th grades)	0.03	0.17	0.04	0.19	0.02	0.14
Some HS or HS graduate	0.30	0.46	0.30	0.46	0.31	0.46
Some college or above	0.66	0.47	0.65	0.48	0.66	0.47
Observations (unweighted)	1032		612		420	

Finally, in terms of the main authoritative school climate predictors in this study, overall, adolescents in the analytic sample had index scores on both disciplinary structure and student support that were approximately a quarter of a standard deviation lower, on average, relative to all students (both victimized and non-victimized). Recall that the index was created using all students and standardized to have a mean of 0. Girls reported lower disciplinary structure index scores relative to boys (−0.33 versus −0.19) while boys reported lower student support (−0.29 versus −0.22).

### Authoritative School Climate and Adolescents’ Outcomes

As shown in Table 2, there were two statistically significant associations between the authoritative school climate predictors and adolescents’ outcomes. Adolescents in schools with higher student support index scores experienced significantly lower odds of reporting negative effects of bullying victimization on their schoolwork (Odds ratio [*OR*] = 0.81; 95% confidence interval [CI] 0.69, 0.95; *p* < 0.05) and negative feelings about themselves (*OR* = 0.84; 95% CI 0.73, 0.98; *p* < 0.05).

**Table 2:** Ordinal regression model results describing the association between authoritative school climate and adolescents’ perception of how negatively bullying victimization impacted their outcomes (2017 School Crime Supplement to the National Crime Victimization Survey; n = 1331)

	Schoolwork		Relationships with friends or family		Feelings about self		Physical health	
Predictors	*OR*	*SE*	*OR*	*SE*	*OR*	*SE*	*OR*	*SE*
Authoritative school climate								
Disciplinary structure index	0.99	(0.12)	0.86	(0.09)	0.88	(0.08)	0.89	(0.11)
Student support index	0.81*	(0.07)	0.86	(0.08)	0.84*	(0.06)	0.92	(0.09)
Victimization type								
Physical victimization	1.20	(0.28)	1.40	(0.29)	1.45	(0.30)	1.23	(0.33)
Social victimization	1.88	(1.28)	3.94	(2.87)	5.84*	(4.02)	2.15	(1.58)
Threatened with harm	1.09	(0.28)	1.30	(0.30)	0.98	(0.23)	1.18	(0.35)
Coercion	1.07	(0.35)	1.84*	(0.56)	1.39	(0.41)	2.09*	(0.69)
Destroy property	1.15	(0.36)	1.07	(0.36)	0.77	(0.26)	1.00	(0.37)
Hate-related words	1.00	(0.22)	0.99	(0.23)	1.14	(0.21)	1.20	(0.30)
Bullying victimization frequency (Reference: One day)								
Two days	1.40	(0.63)	2.04	(0.74)	1.81	(0.56)	2.30	(0.98)
Three to 10 days	6.27***	(2.05)	2.72**	(0.87)	4.59***	(1.29)	3.86***	(1.48)
More than 10 days	10.30***	(3.93)	5.69***	(1.75)	5.92***	(1.64)	5.17***	(2.46)
Fear (factor score)	0.88*	(0.05)	0.90	(0.05)	0.89*	(0.05)	0.88	(0.07)
Avoidance (factor score)	1.43***	(0.13)	1.26**	(0.11)	1.37***	(0.11)	1.27**	(0.12)
Number of school security features	1.04	(0.08)	1.12	(0.09)	1.06	(0.08)	0.93	(0.08)
Caring peer index	0.97	(0.08)	0.93	(0.07)	0.98	(0.08)	1.07	(0.10)
Self-reported grades								
(Reference: C’s or below)								
A’s	0.38***	(0.11)	0.92	(0.23)	0.95	(0.23)	0.39**	(0.11)
B’s	0.62	(0.15)	1.19	(0.28)	1.14	(0.27)	0.50*	(0.14)
Number of extracurricular activities	1.00	(0.11)	1.12	(0.12)	0.93	(0.09)	0.95	(0.10)
Attends a public school	1.09	(0.35)	1.93	(0.94)	0.54	(0.20)	0.78	(0.34)
In middle school (grades 6–8)	1.07	(0.32)	0.98	(0.34)	1.52	(0.43)	1.34	(0.50)
Students brought guns to school	1.27	(0.39)	1.16	(0.41)	1.52	(0.53)	0.65	(0.26)
Involved in physical fights at school	1.27	(0.39)	1.69	(0.55)	0.70	(0.20)	1.17	(0.44)
Gangs at school	0.70	(0.21)	0.55*	(0.14)	0.81	(0.22)	1.12	(0.39)
Age (in years)	0.99	(0.08)	0.99	(0.09)	1.08	(0.08)	0.94	(0.10)
Male	0.85	(0.17)	0.40***	(0.09)	0.59**	(0.11)	0.41**	(0.13)
Race and ethnicity								
(Reference: White, non-Hispanic)								
Black, non-Hispanic	1.07	(0.38)	0.51*	(0.17)	0.79	(0.22)	0.72	(0.23)
Hispanic	1.36	(0.32)	0.91	(0.25)	0.64	(0.15)	0.50*	(0.15)
Asian	2.15	(1.20)	2.78*	(1.36)	2.30	(1.21)	2.03	(1.36)
Another race or ethnicity	0.75	(0.37)	0.57	(0.31)	0.44	(0.22)	0.34	(0.24)
Parental education level								
(Reference: Some college or above)								
Elementary (5th grade or below)	2.09	(2.16)	0.30	(0.20)	0.42	(0.29)	1.36	(0.81)
Middle (6th through 8th grades)	0.70	(0.46)	0.29	(0.22)	1.69	(0.91)	0.54	(0.35)
Some HS or HS graduate	0.94	(0.21)	1.09	(0.21)	0.80	(0.15)	1.09	(0.28)
cut1	3.43^*^	(1.53)	5.15**	(1.70)	4.76**	(1.49)	1.67	(1.92)
cut2	5.49^***^	(1.54)	7.11***	(1.74)	6.27***	(1.50)	3.53	(1.91)
N (unweighted)	1331		1331		1331		1331	

Models include survey weights and design information. Missing data handled through multiple imputation. Taylor linearized standard errors in parentheses

^*^p < 0.05, **p < 0.01, ***p < 0.001

**Fig. 1:**
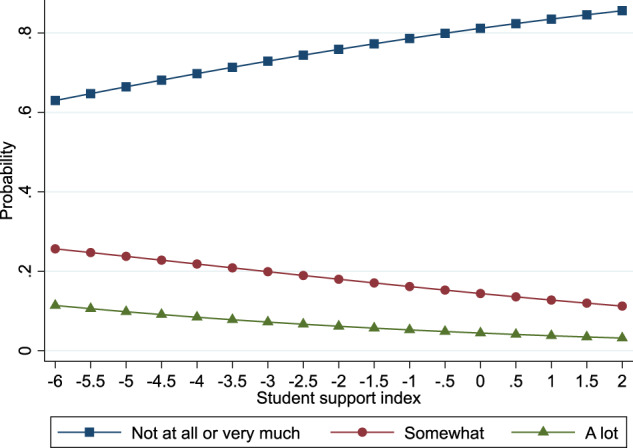
The association between student support and negative feelings about schoolwork after bullying victimization at school

**Fig. 2:**
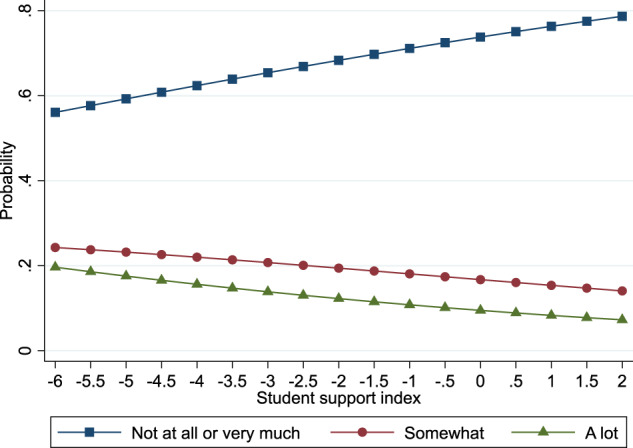
The association between student support and negative feelings of one’s self after bullying victimization at school

Figures 1 and 2 display these results. Figure 1 displays predicted probabilities that bullying victimization negatively impacted adolescents’ schoolwork (a) not at all/not very much, (b) somewhat or (c) a lot according to values on the student support index. As shown, when moving from lower to higher values on the student support index, adolescents experienced a lower probability of reporting that bullying victimization negatively impacted their schoolwork somewhat or a lot. Also, higher levels of student support were associated with higher probabilities of adolescents reporting that bullying victimization did not negatively impact or have very much of an impact on their schoolwork. Similarly, Fig. 2 shows similar changes in the predicted probabilities of experiencing negative feelings about self due to bullying victimization.

Finally, while the results for other outcomes (e.g., relationships with friends and family and physical health) were in the direction that was hypothesized (i.e., a lower odds), this study could not rule out that the associations between authoritative school climate and these other outcomes were zero (*p* > 0.05). Notably, no significant associations were detected between disciplinary structure and each outcome.

### Gender Differences in Response to Authoritative School Climate

Table 3 reports the results of the analyses stratified by gender. Similar to the main results, for female adolescents, student support was significantly associated with lower odds of reporting that bullying victimization negatively influenced their schoolwork (*OR* = 0.74; 95% CI 0.59, 0.95; *p* < 0.05) and negative feelings of self (*OR* = 0.78; 95% CI 0.63, 0.96; *p* < 0.05). In addition to these results, this study also found that for female adolescents, higher levels of disciplinary structure were linked to a lower likelihood of negative feelings about the impact that bullying victimization had on their relationships (*OR* = 0.73; 95% CI 0.56, 0.95; *p* < 0.05). However, as shown in the bottom panel of Table 3 for adolescent boys, this study was unable to detect any statistically significant relationships between authoritative school climate and their outcomes.

**Table 3:** Ordinal regression model results describing the association between authoritative school climate (disciplinary structure and student support) and outcomes of adolescents experiencing victimization. Results stratified by gender (2017 School Crime Supplement to the National Crime Victimization Survey; n_girls_ = 771; n_boys_ = 560)

**Girls**	**Schoolwork**		**Relationships with friends or family**		**Feelings about self**		**Physical Health**	
**Predictors**	***OR***	***SE***	***OR***	***SE***	***OR***	***SE***	***OR***	***SE***
Authoritative school climate								
Disciplinary structure index	0.89	(0.13)	0.73^*^	(0.10)	0.83	(0.09)	0.83	(0.13)
Student support index	0.74^*^	(0.09)	0.82	(0.10)	0.78^*^	(0.08)	0.83	(0.10)
cut1	3.04	(1.87)	5.66^*^	(2.26)	2.85	(1.81)	3.96	(2.64)
cut2	5.15^**^	(1.88)	7.73^***^	(2.28)	4.23^*^	(1.62)	5.84^*^	(2.63)
N (unweighted)	771		771		771		771	
**Boys**	**Schoolwork**		**Relationships with friends or family**		**Feelings about self**		**Physical Health**	
**Predictors**	***OR***	***SE***	***OR***	***SE***	***OR***	***SE***	***OR***	***SE***
Authoritative school climate								
Disciplinary structure index	1.15	(0.20)	1.29	(0.23)	0.95	(0.15)	1.07	(0.25)
Student support index	0.88	(0.11)	0.89	(0.11)	0.91	(0.10)	1.09	(0.19)
cut1	4.52	(2.95)	7.74^*^	(3.13)	9.14^**^	(2.81)	−1.54	(3.68)
cut2	6.67^*^	(2.93)	9.63^**^	(3.24)	10.69^***^	(2.88)	0.54	(3.71)
N (unweighted)	560		560		560		560	

Models include survey weights, design information and controls (results for controls not shown, but available from authors upon request). Missing data handled through multiple imputation. Taylor linearized standard errors in parentheses

*p < 0.05, **p < 0.01, ***p < 0.001

## Discussion

Previous research has shown that authoritative school climate is associated with reductions in bullying victimization (Cornell et al., 2015) and risk behaviors (Cornell & Huang, 2016), but less is known about whether an authoritative climate also offers protective benefits that extend to adolescents’ post-victimization outcomes, including how bullying victimization negatively impacts aspects of their daily lives. This study, based on an analysis of a nationwide sample of adolescents in the United States from the 2017 School Crime Supplement, yielded four new findings. First, there was a significant relationship between the support dimension of authoritative school climate and negative feelings about schoolwork and self after being victimized. Second, disciplinary structure was unrelated to all outcomes tested. Third, these results were driven exclusively by the girls in the sample; no effects were detected for boys. Fourth, for girls, disciplinary structure was related to a reduction in negative feelings about their relationships after being bullied.

Except for the final finding that links disciplinary structure to reduced negative feelings in girls about their relationships, these results contrast with the study’s hypothesis that disciplinary structure would, overall, relate to adolescents’ outcomes. One reason why disciplinary structure may not be related, is the degree to which rules are overly strict. For example, overly strict rules can make students feel demeaned (Lagana-Riordan et al., 2011) and students who perceive their school rules as stricter are more likely to engage in disruptive behaviors (Way, 2011). However, for girls, stronger disciplinary environments could contribute to a stronger sense of security; feeling more secure could mean stronger attachment to and trust of peers at schools, and as a result, this could reduce how negatively they feel about their relationships after being bullied.

The stronger positive effects of adult support on the outcomes of girls is consistent with prior research (Price et al., 2019) and contributes to the literature on gender differences in victimization outcomes. One new contribution of the present study is that the results highlight how support is linked to outcomes that have been previously unaddressed in the authoritative school climate and bullying literature—negative feelings about schoolwork and self. The results also raise the question as to why the effect of adult support is positive for girls but not for boys. One possibility is that girls may experience worse mental health outcomes related to victimization (Hamilton et al., 2015) and thus benefit more from protective buffers than boys. Girls may also have stronger relationships with their teachers (McCormick & O’Connor, 2015), and these relationships could be particularly beneficial if teachers are of the same gender.

Another underlying driver behind these differences could be student support systems that offsets the effects of school structure and benefits one gender but not the other. One such support system consists of empathetic peers, who have been shown to mediate victimization and related psychosocial outcomes (e.g., internalizing and externalizing behaviors, anxiety, and depression) for victimized youth (Rasalingam et al., 2017). For boys, supportive peers could play a more salient role relative to either disciplinary structure or supportive adults while for girls, school structure was more salient. Accordingly, reasons for gender differences in the effects of authoritative climate may depend on other broader school climate features like peers. The salience of peer support relative to adult support is an important distinction and authoritative school climate research could benefit from contextualizing how the effect of disciplinary structure and support interact with other elements of a school’s climate to shape outcomes. Finally, although beyond the scope of this present study, future research can analyze ways in which climate is linked to the wellbeing of additional subgroups of students who experience disproportionate impacts of school bullying victimization, including students with disabilities and those from marginalized racial and ethnic backgrounds (Gage et al., 2021).

The differential effects of authoritative school climate by gender have several policy implications for schools. Given that girls experience greater psychological effects of victimization than boys (Ledwell & King, 2013), this study’s results are promising for schools aiming to reduce this disparity. For example, schools focused on improving girls’ feelings about themselves after victimization should prioritize adult support over disciplinary structure. In addition to providing opportunities for teachers and students to develop stronger relationships, schools should promote teacher professional development that trains teachers to identify when girls may experience victimization and initiate regular check-ins as a follow up. Teachers may also need training to intervene in different types of victimization (Price et al., 2019). In other words, some combination of quick identification and sustained follow up can better support girls after victimization. Of course, other school support staff should also be involved in this process as greater visibility and more engagement with students may both prevent and buffer the effects of bullying victimization. This may entail both structured activities between school adults and students alongside more informal day-to-day interactions.

More broadly, this study directly pinpoints how supportive conditions interwoven throughout the complex climate of schools can shape adolescents’ wellbeing. Since adult support is malleable, it can be enhanced via a shift in policies, practices and norms in order to further support adolescent wellbeing. At the same time, building supportive relationships often needs to move beyond just intentions of support (i.e., referred to as *aesthetic* care, which are “sentimental phrases with little to no action” [p. 11; McHugh et al., 2013]) and instead, focus on authentic engagement. The challenge of building and strengthening authentic support rests largely on adults and educators who know how to establish relationships that respect diversity and deepen collaboration and connection (Cohen et al., 2009). Sustaining support also requires engaging teachers in self-reflective processes and ongoing professional development and training (McHugh et al., 2013). In sum, generating an authentic and student-centered authoritative school climate requires intentionality and commitment.

There are several limitations in this study. First, this study is correlational rather than causal. Future research can leverage quasi-experimental designs to confirm whether authoritative climate is causally linked to these outcomes. Second, this study is unable to pinpoint exactly why support was beneficial while structure, overall, was not. Future studies should leverage qualitative methods that can help capture adolescents’ viewpoints about why supportive and strong disciplinary school environments helped promote their wellbeing despite being victimized at school (Patton et al., 2017). Finally, because this study used cross-sectional data, the findings capture only a brief window in time. More consistent exposure to an authoritative school climate could be influential over time, and thus future research can leverage longitudinal methods to tease out longer term influences.

## Conclusion

A prominent gap in the school victimization literature is how authoritative school climate relates to adolescents’ post-victimization outcomes and whether there are gender differences in that relationship. Prior research has indicated that an authoritative climate can be beneficial for adolescents’ socioemotional adjustment; yet, whether those benefits extend to outcomes after being bullied remains less well understood. This study found that the support dimension helped adolescents feel less negatively about their schoolwork and one’s self after being bullied, a result driven by girls in the sample. No effects were detected for boys. This study underscores the need for ongoing research about and investment in ways to generate and nurture a supportive school climate. Doing so is a collective endeavor, and sustaining such a climate involves a complex interplay of students, teachers, administrators and parents, alongside broader communities, including community engaged stakeholders and researchers. Though challenging, investments in generating a supportive school climate holds considerable promise and potential in transforming schools into places where all students feel safe and protected in the wake of bullying victimization.

## Appendix

**Handling Missing Data**

The amount of missing data varied by each variable (Table 4). Some variables were completed observed while the highest amount of missing data (18%) was for the variable capturing gang presence at school. In total, 25% of the respondents had missing data on one or more variable. Missing data was handled using multivariate imputation by chained equations (MICE) and all variables that had missing data were imputed. In doing so, a set of univariate imputation models was specified for each missing variable. For ordered categorical variables, ordered logistic regression was used, for binary variables, logistic regression and for continuous variables, linear regression. A total of 25 imputed datasets were generated which is equal to the proportion of respondents with incomplete data (White et al., 2011).

**Table 4:** Frequency of missing data on study variables

	Missing values	
	n	%
Negative effects of bullying on:		
Schoolwork	53	3.98
Relationships	55	4.13
Feelings about self	55	4.13
Physical health	55	4.13
Authoritative school climate		
Disciplinary structure index	55	4.13
Student support index	48	3.61
Victimization type		
Physical victimization	52	3.91
Social victimization	50	3.76
Threatened with harm	53	3.98
Coercion	51	3.83
Destroy property	51	3.83
Victimization frequency (10 or more times)	74	5.56
Number of school security features	0	0.00
Caring peer index	67	5.03
Self-reported grades		
Mostly A’s	60	4.51
Mostly B’s	60	4.51
Mostly C’s or below/Other	60	4.51
Number of extracurricular activities	27	2.03
Attends a public school	6	0.45
In middle school (grades 6–8)	0	0.00
Students brought guns to school	56	4.21
Involved in physical fights at school	46	3.46
Gangs at school	239	17.96
Age (in years)	0	0.00
Male	0	0.00
Race and ethnicity		
White, non-Hispanic	0	0.00
Black, non-Hispanic	0	0.00
Hispanic	0	0.00
Asian	0	0.00
Another race/ethnicity	0	0.00
Parental education level		
Elementary (5th grade or below)	0	0.00
Middle (6th through 8th grades)	0	0.00
Some HS or HS graduate	0	0.00
Some college or above	0	0.00

Finally, to check if the imputation models were reasonable, descriptive statistics (means or proportions and standard deviations) were generated for each variable, then pooled across the imputed datasets and, finally, compared those to descriptive statistics on the incomplete data (Table 5). The imputed means and standard deviations are similar to those from the incomplete data demonstrating adequacy of the models.

**Table 5:** Descriptive statistics for the non-imputed and imputed data

	Non imputed		Imputed (n = 25 imputations)	
	Mean or Proportion	*SD*	Mean or Proportion	*SD*
Negative effects of bullying on:				
Schoolwork				
Not at all/Not very much	0.82	0.38	0.80	0.40
Somewhat	0.14	0.34	0.15	0.36
At lot	0.04	0.20	0.05	0.22
Relationships				
Not at all/Not very much	0.82	0.38	0.81	0.39
Somewhat	0.13	0.34	0.14	0.35
At lot	0.05	0.21	0.04	0.21
Feelings about self				
Not at all/Not very much	0.74	0.44	0.73	0.44
Somewhat	0.16	0.37	0.17	0.37
At lot	0.10	0.30	0.10	0.30
Physical health				
Not at all/Not very much	0.87	0.34	0.86	0.35
Somewhat	0.10	0.30	0.11	0.31
At lot	0.03	0.18	0.03	0.18
Authoritative school climate				
Disciplinary structure index	−0.28	1.02	−0.30	0.95
Student support index	−0.25	1.11	−0.24	0.81
Victimization type				
Physical victimization	0.26	0.44	0.27	0.23
Social victimization	0.94	0.24	0.94	0.40
Threatened with harm	0.19	0.39	0.20	0.20
Coercion	0.09	0.29	0.10	0.14
Destroy property	0.07	0.25	0.07	0.12
Hate-related words	0.24	0.43	0.24	0.25
Bullying victimization frequency				
One day	0.31	0.46	0.31	0.46
Two days	0.18	0.39	0.19	0.39
Three to 10 days	0.29	0.45	0.30	0.46
More than 10 days	0.21	0.41	0.21	0.41
Fear (factor score)	−0.28	1.39	−0.36	0.91
Avoidance (factor score)	0.27	1.15	0.30	0.71
Number of school security features	5.99	1.24	5.74	1.62
Caring peer index	−0.13	1.08	−0.17	0.91
Self-reported grades				
Mostly A’s	0.40	0.49	0.38	0.50
Mostly B’s	0.40	0.49	0.41	0.49
Mostly C’s or below/Other	0.20	0.40	0.21	0.35
Number of extracurricular activities	1.12	0.94	1.07	0.93
Attends a public school	0.93	0.26	0.94	0.26
In middle school (grades 6–8)	0.52	0.50	0.51	0.49
Students brought guns to school	0.08	0.27	0.07	0.17
Involved in physical fights at school	0.13	0.34	0.12	0.18
Gangs at school	0.19	0.39	0.18	0.31
Age (in years)	14.30	1.80	14.32	1.89
Male	0.41	0.49	0.43	0.50
Race and ethnicity				
White, non-Hispanic	0.61	0.49	0.59	0.50
Black, non-Hispanic	0.16	0.36	0.15	0.34
Hispanic	0.18	0.38	0.19	0.43
Asian	0.02	0.13	0.03	0.24
Another race or ethnicity	0.04	0.20	0.04	0.19
Parental education level				
Elementary (5th grade or below)	0.01	0.08	0.01	0.15
Middle (6th through 8th grades)	0.03	0.17	0.03	0.20
Some HS or HS graduate	0.30	0.46	0.31	0.46
Some college or above	0.66	0.47	0.65	0.49
Observations (unweighted)	1032		1331	
